# Mitochondria-related miR-151a-5p reduces cellular ATP production by targeting *CYTB* in asthenozoospermia

**DOI:** 10.1038/srep17743

**Published:** 2015-12-02

**Authors:** Ran Zhou, Rong Wang, Yufeng Qin, Juan Ji, Miaofei Xu, Wei Wu, Minjian Chen, Di Wu, Ling Song, Hongbing Shen, Jiahao Sha, Dengshun Miao, Zhibin Hu, Yankai Xia, Chuncheng Lu, Xinru Wang

**Affiliations:** 1 State Key Laboratory of Reproductive Medicine, Institute of Toxicology, Nanjing Medical University, Nanjing 210029, China; 2 Key Laboratory of Modern Toxicology of Ministry of Education, School of Public Health, Nanjing Medical University, Nanjing 210029, China; 3 Research Center for Bone and Stem Cells, Department of Anatomy, Histology, and Embryology, Nanjing Medical University, Nanjing, China; 4 Department of Epidemiology and Biostatistics and Key Laboratory of Modern Toxicology of Ministry of Education, School of Public Health, Nanjing Medical University, Nanjing, China

## Abstract

Mitochondria, acting as the energy metabolism factory, participate in many key biological processes, including the maintenance of sperm viability. Mitochondria-related microRNA (miRNA), encoded by nuclear genome or mitochondrial genome, may play an important regulatory role in the control of mitochondrial function. To investigate the potential role of mitochondria-related miRNAs in asthenozoospermia, we adopted a strategy consisting of initial screening by TaqMan Low Density Array (TLDA) and further validation with quantitative reverse transcriptase polymerase chain reaction (qRT-PCR). Validation of the profiling results was conducted in two independent phases. Eventually, two seminal plasma miRNAs (sp-miRs) (miR-101-3p, let-7b-5p) were found to be significantly decreased, while sp-miR-151a-5p was significantly increased in severe asthenozoospermia cases compared with healthy controls. To further study their potential roles in asthenozoospermia, we then evaluated mitochondrial function of GC-2 cells transfected with these potentially functional miRNAs. Our results demonstrated that transfection with miR-151a-5p mimics decreased the mitochondrial respiratory activity. Besides, Adenosine Triphosphate (ATP) level was decreased when transfected with miR-151a-5p mimics. In addition, Cytochrome b (*Cytb*) mRNA and protein levels were also decreased when miR-151a-5p was overexpressed. These results indicate that miR-151a-5p may participate in the regulation of cellular respiration and ATP production through targeting *Cytb*.

Infertility is a worldwide reproductive health problem which affects approximately 15% of couples, with the man responsible for approximately half of the cases^1^,^2^. Asthenozoospermia, a common cause of male infertility, is characterized by reduced motility or absent sperm motility in the fresh ejaculate, which prevents the sperm from moving to the egg and penetrating it, eventually leading to infertility^3^,^4^. In the process of sustaining human sperm motility, mitochondria plays a pivotal role in providing energy^5^.

Mitochondria, the energy metabolism factory in the cell, participates in generating Adenosine Triphosphate (ATP) through the respiratory chain to provide energy for the cellular activities. The dysfunction of mitochondria has been linked with multiple diseases, including idiopathic asthenozoospermia^6^,^7^,^8^. Mitochondrial function is jointly regulated by the nuclear and mitochondrial genomes. Post-transcriptional regulation of gene expression mediated by microRNAs (miRNAs) is one of the most important regulation mechanism in mitochondrial function.

Recently, several studies have demonstrated that miRNAs, encoded by nuclear genome or mitochondrial genome, not only regulate nuclear genome encoding mitochondria-related proteins, but also could translocate into the mitochondria and regulate mitochondrial genome expression^9^,^10^,^11^,^12^,^13^,^14^. We call these miRNAs, the potential regulators of mitochondrial function, as mitochondria-related miRNAs. Growing evidence implicate mitochondria-related miRNAs in a wide range of biological processes and their dysfunction can lead to diverse diseases^14^,^15^,^16^. In 2008, Aschrafi *et al.* reported that miR-338 decreases mitochondrial oxidative phosphorylation by targeting COXIV mRNA in the Axons of Sympathetic Neurons^15^. MiR-210 induces loss of mitochondrial membrane potential and the apparition of an aberrant mitochondrial phenotype at late stages of Non-Small Cell Lung Cancer^16^. MiR-181c translocates into mitochondria and regulates mitochondrial energy metabolism by targeting mt-COX1 mRNA, which is the product of the mitochondrial genome. Perturbations, induced by MiR-181c, could have important consequences in myocardial pathophysiology^14^. Although mitochondria-related miRNAs have been reported to be associated with multiple diseases, their roles in male infertility, especially asthenozoospermia, are still not clear.

Extracellular miRNAs in the serum, plasma and urine have been shown to be associated with several diseases^17^,^18^,^19^. Some miRNAs can be secreted and delivered into recipient cells, where they can be powerful regulators to modify recipient cells function^20^. Semen is a mixture of spermatozoa and fluid from seminiferous tubules, epididymis, and accessory glands^21^. Seminal plasma miRNAs (sp-miRs) might be generated due to intra-cellular sperm secretion^21^. In our previous study, we have identified some sp-miRs involved in azoospermia^22^. Sp-miRs might be ideal biomarkers of male infertility diagnosis due to their relative ease of access.

To systematically and comprehensively investigate the roles of mitochondria-related miRNAs in semen plasma in asthenozoospermia, we employed a strategy consisting of initial screening by TaqMan Low Density Array (TLDA) and further validation with quantitative reverse transcriptase polymerase chain reaction (qRT-PCR). Validation of the profiling results was conducted in two independent phases. To further study the potential roles of selected differentially expressed sp-miRs involved in asthenozoospermia, we then evaluated mitochondrial function of GC-2 cells transfected with these sp-miRs through testing mitochondrial respiratory activity and ATP production.

## Results

### Detection of mitochondria-related miRNAs on severe asthenozoospermia

**Figure 1:**
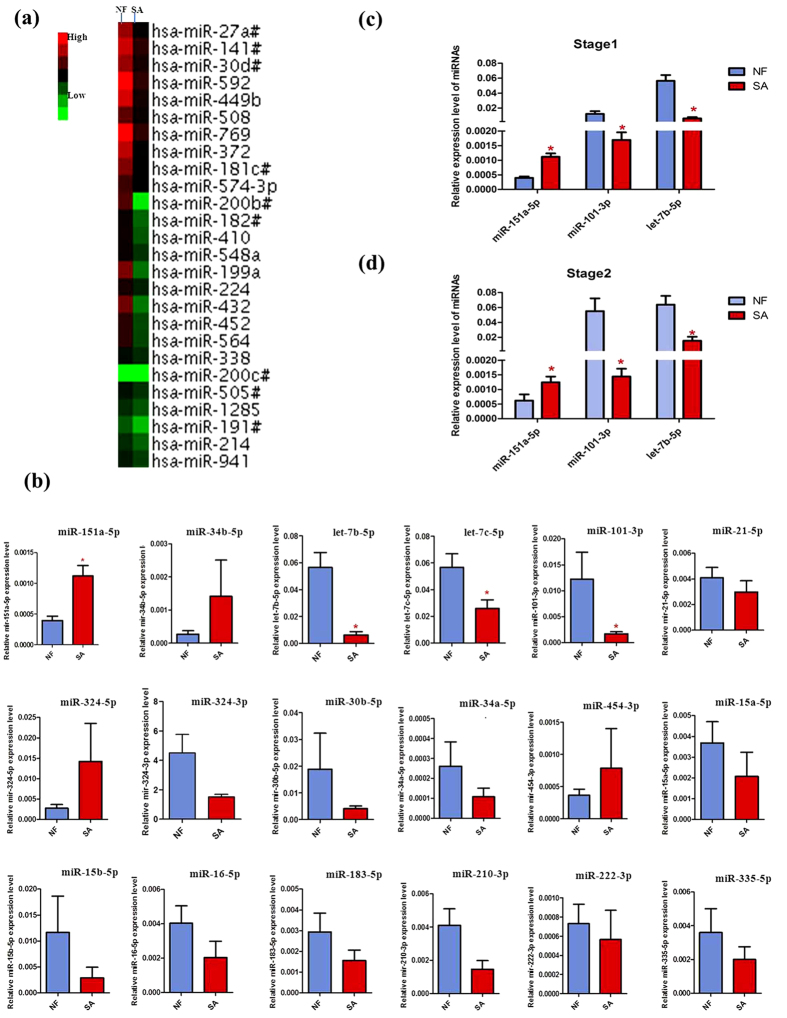
miRNA microarry analysis and qRT-PCR validation of microarry results. (**a**) Heat map representation of the miRNA microarray analysis in normal fertility controls and severe asthenozoospermia using Applied Biosystems Chip. (**b**) qRT-PCR validation of TLDA screening results. (**c**) qRT-PCR validation of stage 1 for microarry results. (**d**) qRT-PCR validation of stage 2 for microarry results. The seminal plasma expression levels of miR-151a-5p,miR-101-3p and let-7b-5p were significantly different between NF and SA (*P* < 0.05). NF, Normal Fertility; SA, Severe Aasthenozoospermia. Asterisks denote significant differences from controls (*P* < 0.05).

To obtain an expression profile of sp-miRNAs that was specific for severe asthenozoospermia, we employed a strategy that included the initial screening by TLDA chips and the validation by qRT-PCR on an individual basis (Fig. 1). We started the search by comparing the miRNA expression profiles of severe asthenozoospermia seminal plasma with those of fertile controls. Of the734 miRNAs screened, 335 and 305 miRNAs were detected by TaqMan miRNA array in the control group and in the severe asthenozoospermia group, respectively. In all subjects, 136 sp-miRNAs were significantly altered, showing 2^−ΔCtcase^/2^−ΔCtcontrol^ >2 or <0.5 in the pooled TLDA chip assay (10 severe asthenozoospermia patients: 10 fertile controls). Based on both scientific and applicability considerations, we selected sp-miRs that had at most a Ct value of 35 by TLDA in both the groups for further individual qRT–PCR confirmation. Then, by referring to a large number of related articles, 18 mitochondria-related miRNAs were selected among the initial differentially expressed miRNAs screening to be further validated (Table 1).

We validated the expression of these 18 sp-miRNAs by qRT–PCR using seminal plasma samples from the same 10 severe asthenozoospermia patients and 10 fertile controls of TLDA screening stage. For each miRNA, we concomitantly tested fertile controls and severe asthenozoospermia patients in the same qRT–PCR plate to minimize intra-plate variations. As shown in Fig. 1b and Supplementary Table S1, the expression levels of four sp-miRs (i.e. miR-151a-5p, miR-101-3p, let-7b-5p, let-7c-5p) were significantly different between the severe asthenozoospermia group and the control group.

Additionally, we validated the expression levels of these four sp-miRNAs in two validation phases using independent populations from Huai’an First people’ Hospital and Wuxi Maternity and Child Health Care Hospital respectively. The seminal plasma expression levels of three sp-miRNAs were significantly different between severe asthenozoospermia patients and fertile controls in the samples of the first validation phase (*P*, 0.0001, 0.0002, and, 0.0001 for miR-151a-5p, miR-101-3p and let-7b-5p, respective) (Fig. 1c), which were repeated in the second validation phase (Fig. 1d).

### MiR-151a-5p decreases mitochondrial respiratory activity

**Figure 2:**
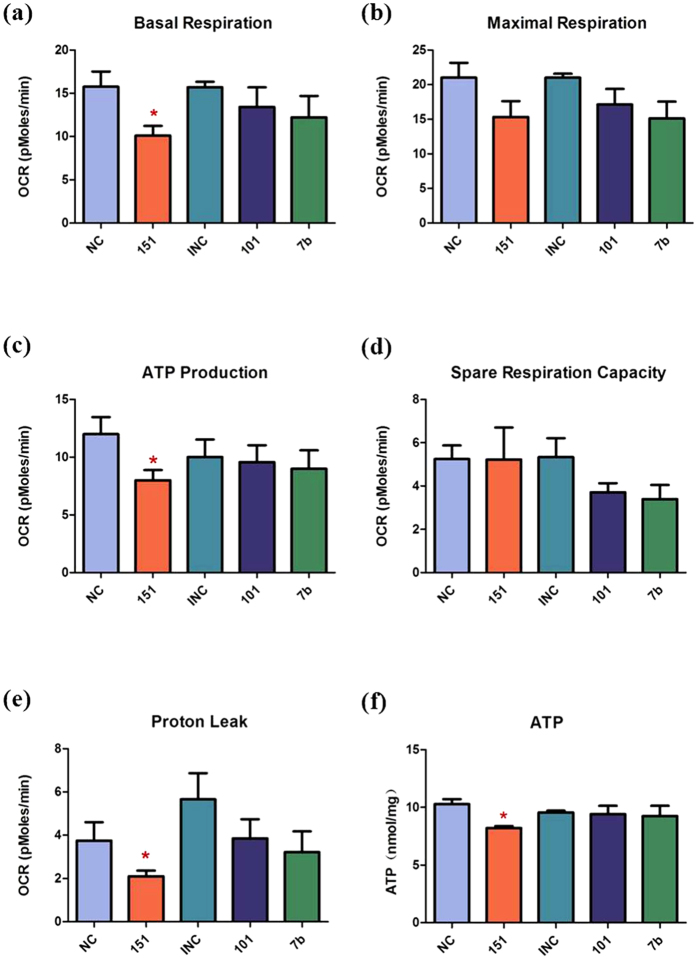
miR-151a-5p causes mitochondrial dysfunction. Compared with NC group, the OCR of basal respiration (**a**), ATP production (**b**), and proton leak (**c**) decreased significantly in the 151 group (*P* < 0.05). There were no differences in maximal respiration (**d**) and spare respiration capacity (**e**) between 151 group and NC group. In 101 and 7b group, there were no differences in above mentioned indexes between these two groups and INC group. (**f**) 151 decreased GC-2 cellular ATP level while other miRNAs (101 and 7b) showed no significantly different. NC, Negative Control; 151, miR-151a-5p; INC, Inhibitor Negative Control; 101, miR-101-3p; 7b, let-7b-5p. Asterisks denote significant differences from controls (*P* < 0.05).

To determine whether these three miRNAs can cause mitochondrial dysfunction, we transfected GC-2 cells with miRNAs using the MitoStress Kit (Seahorse Biosciences) which reflects the activity of electron transport chains in mitochondria directly and monitored the mitochondrial oxygen consumption rate (OCR). Compared with the negative control group, the OCR of basal respiration, ATP production and proton leak decreased significantly in the miR-151a-5p mimics transfection group. However, no significant differences in maximal respiration and spare respiration capacity were found between the miR-151a-5p mimics transfection group and the negative control group. In the miR-101-3p and let-7b-5p inhibition group, no differences were found in the above mentioned indexes between these two groups (Fig. 2a–e). These results indicated that miR-151a-5p might impair mitochondrial function by decreasing mitochondrial respiratory activity.

### MiR-151a-5p decreases cellular ATP level

To ascertain the influence of mitochondrial dysfunction to the level of cellular ATP, we evaluated the cellular ATP level using an ATP detection kit (ATP Assay Kit, Beyotime). We found that miR-151a-5p decreased GC-2 cellular ATP level while the other tested miRNAs (miR-101-3p and let-7b-5p) showed no significant differences (Fig. 2f).

### qRT-PCR for target mRNA and western blot analysis for target protein

**Figure 3:**
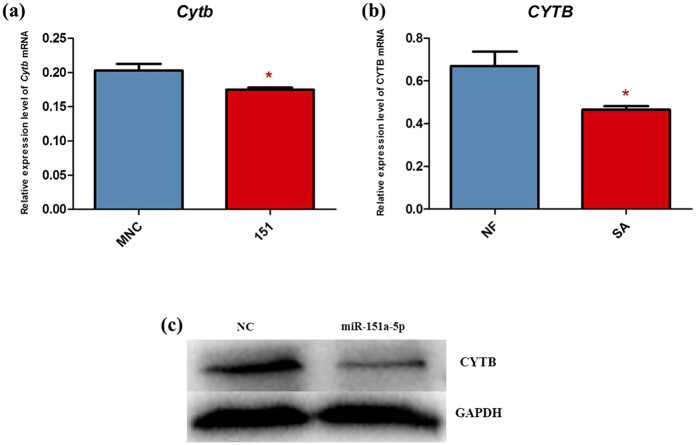
qRT-PCR for *CYTB* mRNA and western blot analysis for CYTB protein. (**a**) Relative expression level of *Cytb* mRNA in MNC and 151 treatment in GC-2 cells. The level of *Cytb* mRNA in 151 treated GC-2 cells was decreased compared with MNC treated GC-2 cells (*P* < 0.05). (**b**) Relative expression level of *CYTB* mRNA in NF and SA. The level of *CYTB* mRNA in SA was decreased compared with NF(*P* < 0.05). (**c**) Western blot analysis for CYTB protein level. The level of CYTB protein in 151 treated GC-2 cells was decreased relative to the MNC group. MNC, Mimics Negative Control; 151, miR-151a-5p; NF, Normal Fertility; SA, Severe Aasthenozoospermia. Full-length blots are presented in Supplementary Figure S1. Asterisks denote significant differences from controls (*P* < 0.05).

In 2011, Barrey *et al.* scanned miRNA targets in the mitochondrial reference sequence (AC_000021.2 GI:115315570) to know if the miRNA silencing machinery could be efficient on some mitochondrial genes. A total of 169 potential targets of miRNAs, including miR-151a-5p, were identified to participate in the modification of mitochondrial function^23^. According to Barrey’s report, *CYTB* mRNA may be a potential target of miR-151a-5p. To test whether miR-151a-5p can influence the expression of *CYTB*, we evaluated *Cytb* mRNA levels in the GC-2 cells transfected with the miR-151a-5p mimics using qRT-PCR mentioned above (primer sequences used are listed in Supplementary Table S2). We found that the level of *Cytb* mRNA (Fig. 3a) were decreased significantly due to the transfection with the miR-151a-5p mimics. Moreover, it falls in line with the results observed in normal fertility and severe asthenozoospermia cases (Fig. 3b). Then we detected the protein level of CYTB using western blot. We found that the level of CYTB protein was decreased relative to the control group (Fig. 3c and Supplementary Figure S1). These results indicate that miR-151a-5p negatively regulates *CYTB* expression at a post-transcriptional level.

## Discussion

Mitochondria participate in various biological processes, including energy production, calcium homeostasis and apoptosis, with the predominant roles differing among mammalian species^5^,^24^,^25^,^26^. Asthenozoospermia is a common cause of male infertility, and its etiology may be related to mitochondrial dysfunction due to its irreplaceable role in ATP generation for sperm motility through the respiratory chain^3^,^4^,^5^. Growing evidence indicates that mitochondrial dysfunction may be associated with post-transcriptional regulation of gene expression by mitochondria-related miRNAs^9^,^10^,^11^,^12^,^13^,^14^.

Extracellular miRNAs, as powerful regulators secreted and delivered into recipient cells, have been reported to be associated with various diseases^17^,^18^,^19^,^20^. Seminal plasma miRNAs(sp-miRs), as one kind of the extracellular miRNAs, might be ideal biomarkers of male infertility diagnosis due to their non-invasiveness and relative ease of access. According to Wang *et al.*, sp-miRs might be derived from intra-cellular sperm secretion^21^.

Therefore, we hypothesize that mitochondria-related miRNAs in seminal plasma may play important roles in asthenozoospermia through regulating mitochondrial function and ATP production.

To test our hypothesis, we firstly screened genome-wide miRNA expression profiling in severe asthenozoospermia cases and healthy fertility controls using the TLDA chips. Then, we selected 18 miRNAs closely related to mitochondrial function based on bioinformatics analysis and mitochondria-related literature (Table 1). Next we performed two independent validation tests and ultimately found miR-151a-5p was significantly increased, while miR-101-3p and let-7b-5p were significantly decreased in seminal plasma of patients with severe asthenozoospermia compared with fertile controls (Fig. 1).

To further investigate the potential roles of these three miRNAs in regulating mitochondrial function and ATP production, we transfected miR-151a-5p, miR-101-3p and let-7b-5p into GC-2 cell lines and tested a series of indexes indicating activity of electron transport chains. Our results showed that OCR of basal respiration, ATP production, and proton leak decreased significantly in the miR-151a-5p mimics transfection group but not in the other two groups (miR-101-3p and let-7b-5p) compared with the negative control group (Fig. 2a–e). These results suggest that miR-151a-5p could induce cellular mitochondrial dysfunction by decreasing activity of mitochondrial electron transport chains. To ascertain miR-151a-5p could decrease cellular ATP level, we measured GC-2 cellular ATP level after transfected with miR-151a-5p mimics, miR-101-3p inhibitors, and let-7b-5p inhibitors respectively. Our results show that miR-151a-5p decreased GC-2 cellular ATP level while the other two miRNAs (miR-101-3p or let-7b-5p) didn’t influence the ATP level (Fig. 2f). Therefore, we inferred that miR-151a-5p could decrease cellular ATP level by regulating mitochondrial electron transport chains.

Several studies have indicated that miR-151a-5p was mainly involved in occurrence and development of tumors and cardiac diseases^27^,^28^,^29^. For instance, miR-151a-5p was reported to be up-regulated in primary breast tumors and its up-regulation can suppress breast tumor cellular metastasis^27^. By directly targeting RhoGDIA, miR-151a-5p can also promote HCC cell invasion and metastasis^28^. In addition, down-regulation of miR-151a-5p contributes to increased susceptibility to arrhythmogenesis during myocardial infarction with estrogen deprivation^29^. Our study is the first to demonstrate that up-regulated miR-151a-5p could also decrease cellular ATP level by regulating mitochondrial electron transport chains in asthenozoospermia.

Moreover, previous studies on mitochondria-related miRNAs mainly focused on studying their roles in regulating mitochondrial pathway of apoptosis^10^,^30^,^31^, mitochondrial metabolism^9^,^11^,^12^,^13^ and ROS production^32^,^33^. This study provided a new insight into the role of mitochondria-related miRNAs in regulating ATP production. In addition, almost all of the previous studies on mitochondria-related miRNAs focused on exploring these miRNAs’ roles in regulating the expression of nuclear-encoded mitochondria-related genes. However, there are few studies on the role of these miRNAs in directly regulating the expression of mitochondrial genome^14^,^34^. Increasing evidence has showed the presence of many miRNAs located in mitochondria^23^,^35^,^36^. According to Barrey’s report, based on the miRNA targets in the mitochondrial reference sequence (AC_000021.2 GI:115315570), *CYTB* was predicted to be the target of miR-151a-5p by using the miRBase target algorithm with a cut-off E-value set to 0.1 (http://microrna.sanger.ac.uk/targets/v5/)^23^. Then, we measured the expression level of *Cytb* in GC-2 cells transfected with miR-151a-5p. Our results show that *Cytb* mRNA and protein levels were reduced in the miR-151a-5p group compared with the negative control group (Fig. 3). These results suggest that miR-151a-5p might negatively regulate the expression of *CYTB* at a post-transcriptional level.

There are five respiratory chain complexes in the inner mitochondrial membrane that jointly generate a proton gradient across the membrane and produce ATP^37^. CYTB, one of the 11 subunits of mitochondrial complex III, is crucial to maintain the function of complex III, and its abnormal expression will disrupt the function of complex III and eventually lead to mitochondrial dysfunction^38^. In this study, our results demonstrate that up-regulated miR-151a-5p decreases ATP production and could cause mitochondrial dysfunction through the targeting of *CYTB*. MiR-151a-5p, potentially affecting the function of mitochondria, deserves comprehensive investigations in order to uncover spermatogenic roles of mitochondria-related miRNAs. Recently, it has been reported that the mitochondrial genome itself encodes abundant small noncoding RNAs^39^. In the future, our aim is to explore the influence of these small noncoding RNAs in idiopathic male infertility, especially asthenozoospermia.

To our knowledge, this is the first systematic study demonstrating the role of mitochondria-related miRNAs in asthenozoospermia. Our results uncover a molecular mechanism by which miR-151a-5p participates in the regulation of the mitochondrial electron transport chain and ATP production by modulating the levels of CYTB, a protein that plays a key role in the assembly of the mitochondrial complex III. This may be related to the molecular etiology of severe asthenozoospermia and suggests a potential application of miR-151a-5p in severe asthenozoospermia therapy.

## Materials and Methods

### Study population

Institutional Ethics Committee of Nanjing Medical University approval was obtained before initiation of this study, and informed consent was obtained from each of the participants included. Routine semen analysis was carried out by light microscopy according to World Health Organization (WHO, 2010) guidelines. All experimental protocol were approved by the Institutional Review Board for Human Studies of Nanjing Medical University.

All severe asthenozoospermia subjects with infertility were selected on the basis of comprehensive andrological examination, including semen analysis, examination of medical history, a series of physical examinations, scrotal ultrasound, hormone analysis, karyotyping and Y chromosome microdeletion filtrate. Furthermore, a questionnaire was used to collect information, including personal background, lifestyle factors, occupational and environmental exposures, genetic risk factors, sexual and reproduction status etc. Those with a history of cryptorchidism, vascular trauma, orchitis, obstruction of the vas deferens, vasectomy, abnormalities in chromosome number or microdeletions of the azoospermia factor region on the Y chromosome were excluded from the study. Semen analysis for sperm concentration, motility and morphology was performed following World Health Organization (WHO) criteria^20^. To ensure the reliability of the diagnosis, each individual was examined twice. All of the control subjects had fathered one or more healthy children without assisted reproductive measures. Overall, 100 subjects (n = 50 for severe asthenozoospermia cases with infertility, n = 50 for fertile controls) were included in this analysis, and the cases were well matched to controls by age, smoking and drinking status. Among these 100 samples, 10 cases and 10 controls seminal plasma samples coming from Nanjing Medical University Affiliated Nanjing Maternity and Child Health Care Hospital were pooled and subjected to TLDA chip screening (human microRNA panel V2.0, Applied Biosystems Inc., CA,USA). Subjects recruited from Huai’an First people’s Hospital (20 cases: 20 controls) and Wuxi Maternity and Child Health Care Hospital (20 cases: 20 controls) were used for the first and second validation phases, respectively.

### Seminal plasma collection and RNA isolation

Seminal plasma was obtained by liquifying and centrifuging semen samples at room temperature within 2 hs after sampling: first liquifying at 37 °C for 30 mins and then centrifuging at 12 000 rpm, 4 °C for 10 mins. The supernatant was carefully removed and stored at −80 °C before biochemical and miRNA analyses.

The total RNA of the seminal plasma, including miRNAs, was extracted with TRIzol Reagent (Invitrogen Life Technologies Co, USA), according to the manufacturer’s instructions. The concentration and purity of RNA were determined by using Nano-Drop^®^ ND-2000, while its quality was verified by denaturing agarose gel electrophoresis.

### Seminal plasma miRNA expression profiling and filtrating of mitochondria-related miRNAs in severe asthenozoospermia

We performed TLDA analysis to identify differentially expressed miRNAs from the two pooled samples (10 severe asthenozoospermia patients versus 10 fertile controls). Megaplex RT reactions and pre-amplification reactions were run according to the manufacturer’s protocol, in which 75 ml of 0.1×TE was added to the PreAmp product and a 9 ml diluted PreAmp product was used to run the qRT-PCRs by dispensing 100 ml of the PCR reaction mix into each port of the TaqMan MicroRNA array. The pre-amplified product was loaded into TaqMan Array Human Micro-RNA A + B Cards Set v2.0 (Applied Biosystems, CA, USA) enabling simultaneous quantitation of 734 human miRNAs. TaqMan MicroRNA assays were performed on the ABI 7900HT instrument (Applied Biosystems). U6 snRNA (RNU6B; Applied Biosystems) served as an endogenous control. The default PCR procedure was used, and the analysis was performed by using the RQ manager software (Applied Biosystems). ΔCt was calculated using the following mathematical formula: ΔCt = C_tsample_ − C_tU6_. Then, by referring to a large number of literature, we filtrated those miRNAs closely related to mitochondrial function from differential expressed miRNAs above.

### qRT-PCR of mature miRNA

In the validation phases, candidate miRNAs identified by TLDA and filtration were further characterized. Complementary DNA (cDNA) was synthetized from total RNA using miRNA-specific primers. Total RNA (500 ng) was reverse-transcribed using PrimeScript® RT Master Mix (Takara) reverse transcriptase according to the manufacturer’s instructions. Real-time PCR analyses were performed with FastStart Universal SYBR-Green Master (Roche Diagnostics). RNU6B (Applied Biosystems) served as an endogenous control. A quantitative real-time PCR was performed in triplicate using an ABI Prism 7900HT (Applied Biosystems). Cycling conditions were as follows: incubation at 50 °C for 2 min, denaturation at 95 °C for 10 min, followed by 40 cycles of denaturation for 15 s at 95 °C and annealing and extension for 1 min at 60 °C. Fluorescent data were converted to Ct measurements by the 7900 SDS system software (Version 2.4; Applied Biosystems). We assigned an equal number of patients and controls on one plate and run the qPCR for target miRNAs and RNU6B simultaneously. The mean Ct value was determined from three PCR replicates. The amount of target miRNAs was normalized relative to the amount of RNU6B (ΔCt = C_tsample_ − C_tU6_). MiRNA-specific primer sequences used are listed in Supplementary Table S3.

### Cell culture, transient transfection of mimics or inhibitors

GC-2 cells were seeded in 24-well plates and cultured in Dulbecco’s modified Eagle’s medium with 10% fetal bovine serum at 37 °C under 5% CO_2_ atmosphere. Cells were cultured to ~50% confluence and transfection was carried out using Lipofectamine 2000 (Invitrogen) with 50 nM of sp-miR mimics(small, chemically modified double-stranded RNAs that mimic endogenous miRNAs) or a scrambled negative control(Gene Pharma) and 100 nM of sp-miRNA inhibitors(small, chemically modified single-stranded RNA molecules designed to specifically bind to and inhibit endogenous miRNA molecules) or a scrambled negative control (Gene Pharma) according to the manufacturer’s instructions. Cells were harvested at 48 h after transfection.

### Measurements of cellular oxygen consumption rate

The Seahorse XF96 Extracellular Flux Analyzer (Seahorse Biosciences, North Billerica, MA) was used to obtain real-time measurements of oxygen consumption rate (OCR) in GC-2 cells. The Mito Stress Test Kit (Seahorse Biosciences) was used to measure basal respiration, ATP production, proton leak, maximal respiration and spare respiration capacity. The preparation of cells and analysis for the assay was performed according to the manufacturer’s instructions.

### ATP measurements

After being transfected for 48 h, cells were collected to 1.5- mL tube and centrifuged to the bottom. Then, cells were washed with phosphate buffer saline (PBS) twice. Next, 200 μL lysis buffer from ATP Assay kit was add to each tube and then ultrasonicated. The lysate were centrifuged at 12 000 rpm for 5 min at 4 °C. The supernatant was transferred to a new 1.5-mL tube for ATP test with the ATP detection kit purchased from Beyotime. The protein level of the supernatant was measured at 562 nm with Bicinchoninic Acid assay (Beyotime). The relative ATP level was calculated according to the following formula: relative ATP level = ATP value/protein value.

### miRNA target predictions

In order to investigate the potential target genes of miRNAs, we employed a combined target prediction strategy including using *in silico* prediction algorithms by Sanger miRBase (http://www.microrna.sanger.ac.uk/) and TargetScan (http://www.targetscan.org/) and referred to related literature^23^.

### Western blot analysis of target protein

Cells were lysed with RIPA lysis buffer (1% Triton X-100, 50 mM Tris-HCL, 135 mM NaCl, 0.1% sodium deoxycholate, 2 mM EDTA, 50 mM NaF, 2 mM sodium orthovanadate, 10 ug/ml of aprotinin, 10 ug/ml of leupeptin and 1 mM PMSF). Protein lysates were loaded onto SDS/PAGE gels and transferred to nitrocellulose membranes. Blots were blocked in 5% nonfat dry milk dissolved in PBS and incubated with specific antibodies overnight. Antibodies used in the present study were Anti-MT-CYB antibody (1:1000; abcam), anti-GAPDH antibody (1:1000, Beyotime). Protein expression was visualized with an ECL plus kit(Millipore Corporation, Billerica, MA, USA).

### Statistical analysis

miRNA data are presented as the means ± S.E. Statistical comparisons were performed using unpaired two-tailed Student’s t test. All statistical analyses were carried out using IBM SPSS Version 20.0, and *P* < 0.05 was considered to be significant.

## Additional Information

**How to cite this article**: Zhou, R. *et al.* Mitochondria-related miR-151a-5p reduces cellular ATP production by targeting *CYTB* in asthenozoospermia. *Sci. Rep.* **5**, 17743; doi: 10.1038/srep17743 (2015).

## Supplementary Material

Supplementary Information

## Figures and Tables

**Table 1:** Eighteen mitochondria-related miRNAs selected from severe asthenozoospermia.

miRNA	Fold change	Regulation	Sequence	Chr	Target gene/Target gene predicted
miR-151a-5p	8.2738	up	UCGAGGAGCUCACAGUCUAGU	Chr8	*CYTB*^23^
miR-34b-5p	2.4916	up	UAGGCAGUGUCAUUAGCUGAUUG	Chr11	*ATP8*, *COX2*^23^
let-7b-5p	2.0367	down	UGAGGUAGUAGGUUGUGUGGUU	Chr22	*ATP6*, *ATP8*, *COX2*, *ND5*^23^
let-7c-5p	7.8409	down	UGAGGUAGUAGGUUGUAUGGUU	Chr21	*ATP6*, *ATP8*, *COX2*^23^
miR-101-3p	2.0692	down	UACAGUACUGUGAUAACUGAA	Chr1	*ATP5B*^9^, *Mcl-1*^10^
miR-21-5p	3.7405	down	UAGCUUAUCAGACUGAUGUUGA	Chr17	*ND1*^23^
miR-324-3p	3.9598	down	ACUGCCCCAGGUGCUGCUGG	Chr17	*ND6*^23^
miR-324-5p	4.7185	down	CGCAUCCCCUAGGGCAUUGGUGU	Chr17	*ND6*^23^
miR-30b-5p	4.0156	down	UGUAAACAUCCUACACUCAGCU	Chr8	*ND6*^23^
miR-34a-5p	8.8067	down	UGGCAGUGUCUUAGCUGGUUGU	Chr1	*Txnrd2*^32^
miR-454-3p	8.3649	down	UAGUGCAAUAUUGCUUAUAGGGU	Chr17	*COX1*^23^
miR-15a-5p	2.4588	down	UAGCAGCACAUAAUGGUUUGUG	Chr13	*UCP-2*^40^, *Bcl-2*^41^
miR-15b-5p	3.3560	down	UAGCAGCACAUCAUGGUUUACA	Chr3	*Arl2*^12^, *Bcl-2*^42^
miR-16-5p	2.1482	down	UAGCAGCACGUAAAUAUUGGCG	Chr13/Chr3	*Bcl-2*^42^
miR-183-5p	2.6555	down	UAUGGCACUGGUAGAAUUCACU	Chr7	*IDH2*^11^
miR-210-3p	2.6801	down	CUGUGCGUGUGACAGCGGCUGA	Chr11	*SDHD*^16^, *COX10*^13^
miR-222-3p	4.2907	down	AGCUACAUCUGGCUACUGGGU	ChrX	*PUMA*^30^
miR-335-5p	3.5042	down	UCAAGAGCAAUAACGAAAAAUGU	Chr7	*SOD2*^32^
