# Supplementary material for: Mitochondria-related miR-151a-5p reduces cellular ATP production by targeting CYTB in asthenozoospermia
Source: Sci Rep. 2015 Dec 2;5:17743. doi: 10.1038/srep17743 (PMC4667214; doi:10.1038/srep17743)
Supplement: Supplementary Information [file srep17743-s1.doc]

**Mitochondria-related miR-151a-5p reduces cellular ATP production by targeting *CYTB* in asthenozoospermia**

**Ran Zhou^1, 2, †^, Rong Wang^3, †^, Yufeng Qin^1, 2, †^, Juan Ji^1, 2^, Miaofei Xu^1, 2^, Wei Wu^1, 2^, Minjian Chen^1, 2^, Di Wu^2^, Ling Song^2^, Hongbing Shen^1, 4^, Jiahao Sha^1^, Dengshun Miao^1, 3^, Zhibin Hu^1, 4^, Yankai Xia^1, 2^, Chuncheng Lu^1, 2^,** ^*^ **and Xinru Wang^1, 2^,** ^*^

**Table S1. Results of 18 miRNAs in the validation of TLDA results phase**.

| miRNA | Severe asthenozoospermia | | Fertile control | | 2^-△Ct case^/2^-△Ct control b^ | *P* ^c^ |
| --- | --- | --- | --- | --- | --- | --- |
|  | N | mean±SE^a^ | N | mean±SE^a^ |  |  |
| miR-151a-5p | 10 | 0.001119±0.0001717 | 10 | 0.0003937±0.00007276 | 8.276 | 0.001 |
| miR-34b-5p | 10 | 0.001412±0.001098 | 10 | 0.0002699±0.0001056 | 2.492 | 0.397 |
| let-7b-5p | 10 | 0.006293±0.002554 | 10 | 0.05644±0.01116 | 0.491 | 0.002 |
| let-7c-5p | 10 | 0.02582±0.006444 | 10 | 0.05653±0.01031 | 0.128 | 0.034 |
| miR-101-3p | 10 | 0.001690±0.0003835 | 10 | 0.01225±0.005200 | 0.483 | 0.008 |
| miR-21-5p | 10 | 0.002974±0.0008760 | 10 | 0.004085±0.0008092 | 0.267 | 0.372 |
| miR-324-3p | 10 | 0.001499±0.001920 | 10 | 0.004494±0.001267 | 0.253 | 0.093 |
| miR-324-5p | 10 | 0.01423±0.009340 | 10 | 0.002760±0.0009144 | 0.212 | 0.189 |
| miR-30b-5p | 10 | 0.004108±0.001003 | 10 | 0.01882±0.01357 | 0.249 | 0.662 |
| miR-34a-5p | 10 | 0.0001077±0.00004323 | 10 | 0.0002604±0.0001231 | 0.114 | 0.101 |
| miR-454-3p | 10 | 0.0007859±0.0006179 | 10 | 0.0003669±0.00009442 | 0.035 | 0.354 |
| miR-15a-5p | 10 | 0.002073±0.001168 | 10 | 0.003685±0.001022 | 0.407 | 0.329 |
| miR-15b-5p | 10 | 0.002861±0.002083 | 10 | 0.01167±0.007008 | 0.298 | 0.081 |
| miR-16-5p | 10 | 0.002033±0.0009548 | 10 | 0.004038±0.001006 | 0.465 | 0.173 |
| miR-183-5p | 10 | 0.001556±0.0005122 | 10 | 0.002942±0.0009060 | 0.377 | 0.230 |
| miR-210-3p | 10 | 0.001461±0.0005224 | 10 | 0.004111±0.0009867 | 0.373 | 0.076 |
| miR-222-3p | 10 | 0.0005655±0.0003043 | 10 | 0.0007306±0.0002025 | 0.233 | 0.659 |
| miR-335-5p | 10 | 0.001994±0.0007568 | 10 | 0.003598±0.001415 | 0.285 | 0.305 |

^a^ relative expression values are given as mean±standard error(SE).

^b^ 2^-△Ct case^/2^-△Ct control^ from TLDA data.

^c^ unpaired two-tailed Student’s t test from individual assay data.

**Table S2. Target gene primer sequences**.

| **Gene** | **Direction** | **Prmier sequence (5’-3’)** | **Direction** | **Prmier sequence (5’-3’)** |
| --- | --- | --- | --- | --- |
| *GAPDH* | Forword | GCACCGTCAAGGCTGAGAAC | Reverse | GGATCTCGCTCCTGGAAGATG |
| *CYTB* | Forword | GAAGGGCAAGATGAAGTGAAAG | Reverse | TTACTATCCGCCATCCCATAC |

**Table S3. MiRNA-specific primer sequences for Real-time quantitative PCR in the validation phases** .

| **miRNA** | **Direction** | **Prmier sequence (5’-3’)** | **Direction** | **Prmier sequence (5’-3’)** |
| --- | --- | --- | --- | --- |
| miR-151a-5p | RT^a^ | GTCGTATCCAGTGCAGGGTCCGAGGTATTCGCACTGGATACGACACTAGA | Forward | GCGGCGGTCGAGGAGCTCACAG |
| miR-34b-5p | RT^a^ | GTCGTATCCAGTGCAGGGTCCGAGGTATTCGCACTGGATACGACCAATCA | Forward | GCGGCGGTAGGCAGTGTCATTAG |
| let-7b-5p | RT^a^ | GTCGTATCCAGTGCAGGGTCCGAGGTATTCGCACTGGATACGACAACCAC | Forward | GCGGCGGTGAGGTAGTAGGTTG |
| let-7c-5p | RT^a^ | GTCGTATCCAGTGCAGGGTCCGAGGTATTCGCACTGGATACGACAACCAT | Forward | GCGGCGGTGAGGTAGTAGGTTG |
| miR-101-3p | RT^a^ | GTCGTATCCAGTGCAGGGTCCGAGGTATTCGCACTGGATACGACTTCAGT | Forward | GCGGCGGTACAGTACTGTGATAAC |
| miR-21-5p | RT^a^ | GTCGTATCCAGTGCAGGGTCCGAGGTATTCGCACTGGATACGACTCAACA | Forward | GCGGCGGTAGCTTATCAGACTG |
| miR-324-3p | RT^a^ | GTCGTATCCAGTGCAGGGTCCGAGGTATTCGCACTGGATACGACCCAGCA | Forward | GCGGCGGACTGCCCCAGGTGCTG |
| miR-324-5p | RT^a^ | GTCGTATCCAGTGCAGGGTCCGAGGTATTCGCACTGGATACGACACACCA | Forward | GCGGCGGCGCATCCCCTAGGGC |
| miR-30b-5p | RT^a^ | GTCGTATCCAGTGCAGGGTCCGAGGTATTCGCACTGGATACGACAGCTGA | Forward | GCGGCGGTGTAAACATCCTACAC |
| miR-34a-5p | RT^a^ | GTCGTATCCAGTGCAGGGTCCGAGGTATTCGCACTGGATACGACACAACC | Forward | GCGGCGGTGGCAGTGTCTTAGC |
| miR-454-3p | RT^a^ | GTCGTATCCAGTGCAGGGTCCGAGGTATTCGCACTGGATACGACACCCTA | Forward | GCGGCGGTAGTGCAATATTGC |
| miR-15a-5p | RT^a^ | GTCGTATCCAGTGCAGGGTCCGAGGTATTCGCACTGGATACGACCACAAA | Forward | GCGGCGGTAGCAGCACATAATG |
| miR-15b-5p | RT^a^ | GTCGTATCCAGTGCAGGGTCCGAGGTATTCGCACTGGATACGACTGTAAA | Forward | GCGGCGGTAGCAGCACATCATG- |
| miR-16-5p | RT^a^ | GTCGTATCCAGTGCAGGGTCCGAGGTATTCGCACTGGATACGACTGTAAA | Forward | GCGGCGGTAGCAGCACGTAAAT |
| miR-183-5p | RT^a^ | GTCGTATCCAGTGCAGGGTCCGAGGTATTCGCACTGGATACGACAGTGAA | Forward | GCGGCGGTATGGCACTGGTAG |
| miR-210-3p | RT^a^ | GTCGTATCCAGTGCAGGGTCCGAGGTATTCGCACTGGATACGACTCAGCC | Forward | GCGGCGGCTGTGCGTGTGACAG |
| miR-222-3p | RT^a^ | GTCGTATCCAGTGCAGGGTCCGAGGTATTCGCACTGGATACGACACCCAG | Forward | GCGGCGGAGCTACATCTGGCTAC |
| miR-335-5p | RT^a^ | GTCGTATCCAGTGCAGGGTCCGAGGTATTCGCACTGGATACGACACCCAG | Forward | GCGGCGGTCAAGAGCAATAACG |
|  | URP^b^ | ATCCAGTGCAGGGTCCGAGG | | |
| U6 | RT^a^ | CTCAACTGGTGTCGTGGA | Forward | CTC GCT TCG GCA GCA CA |
| U6 | Reverse | | | AAC GCT TCA CGA ATT TGC GT |

^a^ Rreverse Transcription

^b^ Universal Reverse Primer

**
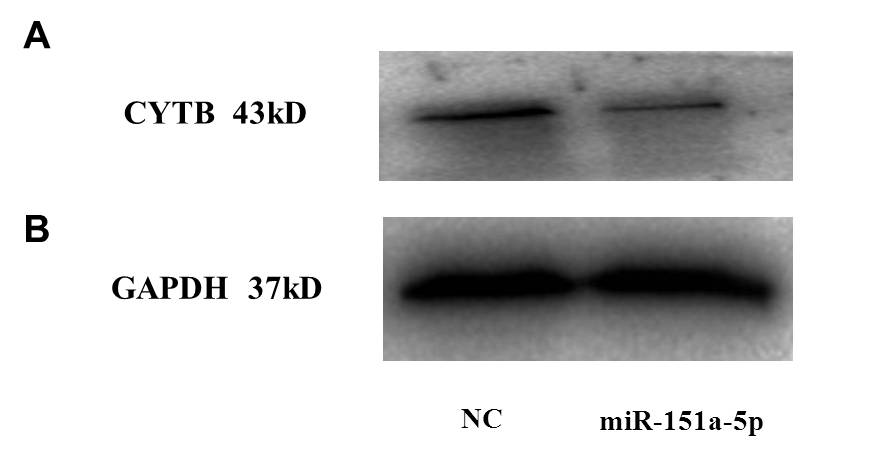
**

**Figure S1.** Western blot analysis for CYTB protein level. The level of CYTB protein in 151 treated GC-2 cells was decreased relative to the NC group.
